# Domestic Cat Hepadnavirus Infection in Iberian Lynxes

**DOI:** 10.3201/eid3101.240568

**Published:** 2025-01

**Authors:** Georgia Diakoudi, Sabrina Castro-Scholten, Javier Caballero-Gómez, Barbara Di Martino, Federica Di Profio, Vittorio Sarchese, Francesco Pellegrini, Gianvito Lanave, Nicola Decaro, Ignacio García-Bocanegra, Vito Martella

**Affiliations:** University of Bari Aldo Moro, Bari, Italy (G. Diakoudi, F. Pellegrini, G. Lanave, N. Decaro, V. Martella); University of Córdoba, Córdoba, Spain (S. Castro-Scholten, J. Caballero-Gómez, I. García-Bocanegra); Centro de Investigación Biomédia en Red–Intermedadaes Infecciosas, Instituto de Salud Carlos III, Madrid, Spain (J. Caballero-Gómez, I. García-Bocanegra); University of Teramo, Italy (B. Di Martino, F. Di Profio, V. Sarchese); University of Veterinary Medicine, Budapest, Hungary (V. Martella)

**Keywords:** Domestic cat hepadnavirus, Iberian lynx, genotype, genome, antibodies, core, surface, viruses, Iberian Peninsula, Italy, Spain, hepatitis B virus

## Abstract

We conducted a survey for domestic cat hepadnavirus, an analog of human hepatitis B virus, in the endangered felid species Iberian lynx. Results revealed specific antibodies in 32.3% of serum samples and DNA in 0.5% of available liver samples. Phylogenetically, the virus segregated apart from other Europe strains of the virus.

Domestic cat hepadnavirus (DCH) is a novel member of the genus *Orthohepadnavirus*, family *Hepadnaviridae*, similar to the prototype species hepatitis B virus (HBV). The virus was first documented in 2018 in Australia in a domestic cat with lymphoma; since then, the virus has been described in cats all over the world (*1*,*2*). The DCH genome is a circular, partially double-stranded DNA, ≈3.2 kb in length, containing 2 large and 2 smaller open reading frames, encoding for the surface protein, the polymerase protein, the precore/core protein, and the X protein (*1*).

HBV infection is a global health challenge representing a major cause of chronic liver diseases in humans, including cirrhosis and hepatocellular carcinoma (*2*). Similarly, reports have correlated DCH with development of feline liver disease and identified the virus in cats with chronic hepatitis and cats with hepatocellular carcinoma (*2*–*4*), stimulating research to investigate the possible implications for feline health. Researchers have reported the virus, at a very low prevalence, also in dogs (*5*); however, studies assessing the susceptibility of other animal hosts to DCH or DCH-like viruses remain elusive.

The Iberian lynx (*Lynx pardinus*) is the most endangered felid species in the world (*6*). By the early 21st Century, Iberian lynx population was estimated to include 156 adult animals in Portugal and Spain (*6*). In response to those findings, conservation organizations launched projects focusing on both in situ and ex situ conservation programs, one of which was the European Commission’s EU LIFE-Nature and Biodiversity programme (*7*). Because of such efforts, the Iberian lynx census has increased considerably during the past decade, reaching >1,600 free-ranging lynxes in 2022. Amid the conservation activities surrounding this species emerged investigations into the pathogens that could pose threats to these animals, such as SARS-CoV-2 and feline leukemia virus (*8*,*9*). We investigated the exposure of Iberian lynxes to DCH.

## The Study

We performed a survey on liver samples collected from 191 Iberian lynxes subjected to necroscopy in 2017–2023 throughout the Iberian Peninsula. Our screening also included 103 serum samples obtained from 100 lynxes affiliated with health programs in a 14-year time frame spanning 2010–2023. Both liver and serum samples were available for 7 lynxes. We obtained all samples from serum and tissue banks at the Center for Analysis and Diagnosis of Wildlife (Andalusia, Spain) and stored them at −80°C before shipment to the Department of Veterinary Medicine, University of Bari (Bari, Italy) for the analyses. We homogenized (10% wt/vol) liver tissues in Dulbecco modified Eagle’s medium and extracted viral DNA from the supernatant of the homogenates and from the serum by using the IndiSpin Pathogen Kit (Indical Bioscience GmbH, https://www.indical.com). We screened DNA extracts for the presence of DCH by using a quantitative PCR (*10*) and a qualitative PCR with panhepadnavirus primers targeting the polymerase ORF (*11*).

Our analyses revealed 1 (0.5%) of the 191 liver samples testing positive for viral DNA by qualitative PCR; none of the serum samples contained viral DNA. We traced the DCH-positive sample to a 7-year-old male Iberian lynx, raised in captivity in Andalusia in southern Spain (collection date March 2021). Samples for the animal included 4 serum samples collected over a 5-year period, 2016–2020; screening showed DCH DNA in only the fourth serum sample (collection date December 2020). We performed DNA enrichment for the DCH-positive liver sample (SPA/2022/Iberian lynx/296-23-81 strain) by using a rolling circle amplification technique with a TempliPhi 100 amplification kit (GE Healthcare, https://www.gehealthcare.com). We used the amplification product as a template for amplifying DCH genome fragments in PCR. A total of 500 ng of equimolar pooled PCR products made up the input for a library prepared using the Ligation Sequencing Kit V14 (Oxford Nanopore Technology, https://nanoporetech.com), according to manufacturer’s guidelines. We performed sequencing by using flongle flow cell R10.4.1 adapted in the MinION Mk1C platform (Oxford Nanopore Technology) for 24 hours.

We generated the complete DCH genome of the SPA/2022/Iberian lynx/296-23-81 strain (GenBank accession no. PP347721) measuring 3,184 bp in length. The Iberian lynx strain displayed 98.3% nucleotide identity to the Thailand strain CP87H_THA/2019 (GenBank accession no. MT506044) and <96.5% nucleotide identity to other DCH strains from Europe. On phylogenetic analysis, the strain SPA/2022/Iberian lynx/296-23-81 segregated with Thailand DCH strains within genotype A, into the distinct subtype A3, apart from other DCH strains from Europe, which segregated within either subtype A1 or subtype A2 (Figure).

**Figure:**
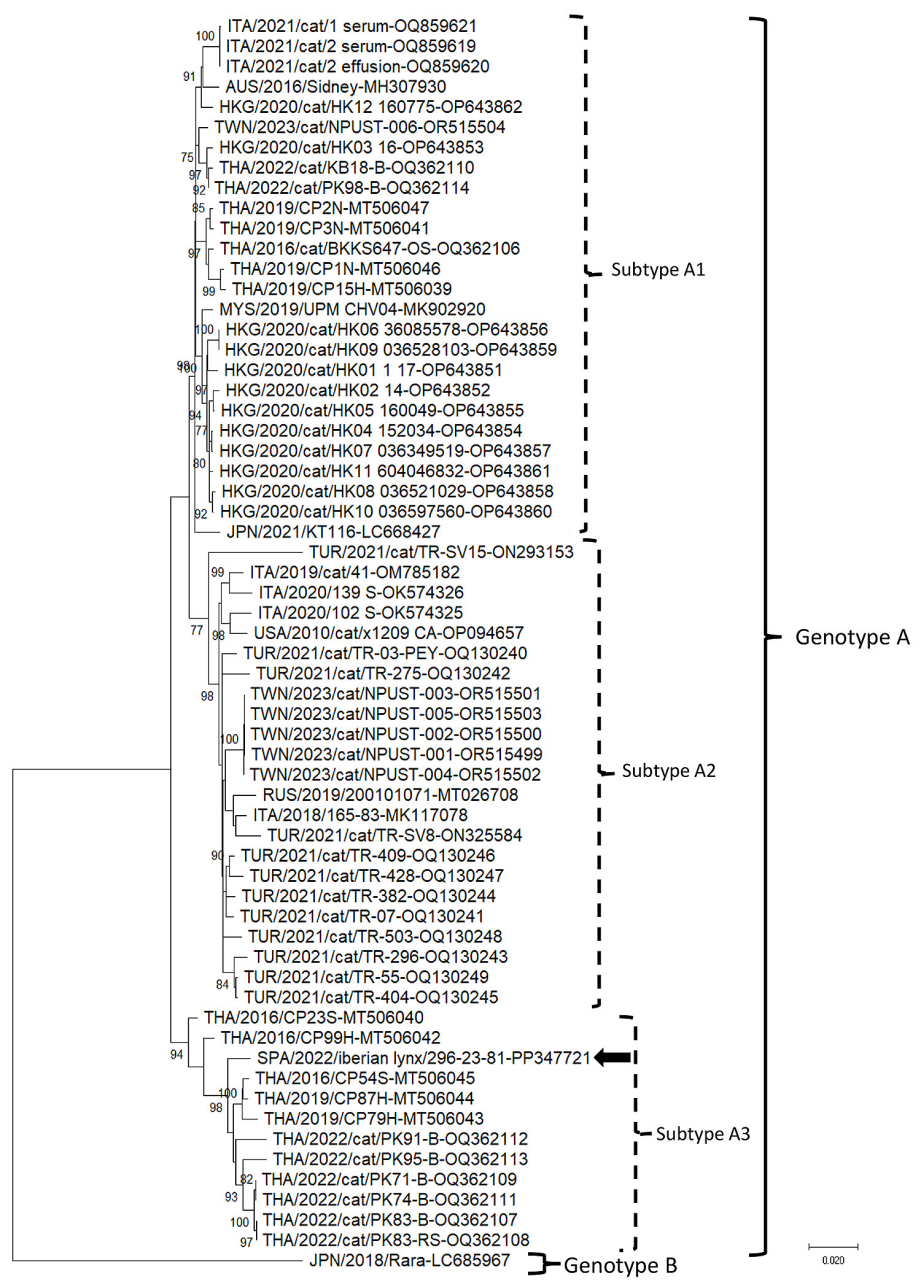
Neighbor-joining phylogenetic tree based on the complete genome of domestic cat hepadnavirus (DCH) from a study of DCH infection in Iberian lynxes. We elaborated the tree by using the alignment of the full-length nucleotide sequence of the Iberian lynx DCH SPA/2022/Iberian lynx/296-23-81 strain (black arrow; GenBank accession no. PP347721) generated in this study and the cognate sequences of DCH strains retrieved from GenBank (accession numbers shown). We constructed the tree by using the maximum-likelihood method, the Kimura 2-parameter model, a discrete gamma distribution and a proportion of invariant sites, and bootstrapping up to 1,000 replicates. Bootstrap values >70% are shown. We used the Japanese Rara strain (GenBank accession no. LC685967), belonging to DCH genotype B, as an outgroup. Scale bar indicates number of nucleotide substitutions per site.

We tested all serum samples at a dilution of 1:100 by using 2 in-house ELISA assays, one based on the recombinant core (DCHcAg) antigen and one based on the surface (DCHsAg) antigen (*12*,*13*), to evaluate the serologic response against DCH. The 4 serum samples collected at different points from the DCH-positive Iberian lynx reacted for DCHcAg IgG but not for DCHcAg IgM or DCHsAg IgG. Our testing detected viral DNA only in the last serum sample from the animal. That pattern is consistent with the status of HBV reactivation, characterized by a peak of viremia in persons with inactive infection, wherein the virus is barely detectable in the serum although replicating in the liver (*3*). Our analysis also revealed DCHcAg IgG in 32 (32.3%) of 99 serum samples, with the highest detection rate in adult free-ranging lynxes (7/16, 43.8%), and no DCHcAg IgM. Only 10 (31.2%) of the 32 serum samples with DCHcAg IgG had also DCHsAg IgG, suggesting clearance from the infection. In humans, HBVcAg IgG is persistent and indicative of exposure to HBV, regardless of the evolutive stage of the infection (*14*).

## Conclusions

Our study results provide evidence for a wide circulation of DCH in the Iberian lynx population, with a seroprevalence rate (32.3%) higher than those observed in cats (25%) and dogs (10%) in Italy (*12*,*13*). We propose the need for additional studies to assess the effect of this virus on the health status of the Iberian lynx. Because cats are considered the primary reservoir of feline leukemia virus infection for the lynx population (*9*), it will be specifically important to investigate the role of domestic cats as a potential source of DCH infection for lynxes.

DCH appears to follow a pattern similar to that of HBV, presenting different types and subtypes based on nucleotide sequence diversity. In HBV, genotypes and subgenotypes might even play a crucial role in clinical outcomes, influencing disease evolution and drug resistance (*15*). In our study, the Iberian lynx DCH strain did not segregate phylogenetically with other DCH strains from Europe detected in cats, raising questions as to the epidemiology of DCH and whether DCH subtype A3 exists in feline populations across the Iberian Peninsula or whether it is a hallmark of the Iberian lynx population. As more clinical and epidemiologic research on DCH unfolds, so might a greater understanding of whether different DCH types and subtypes exhibit phenotypic variations.

The viromes of closely related animal species, or even of species more distant in the evolutionary scale, are largely interconnected, with repeated events of interspecies transmissions and several examples of successful adaptation. Still, the patterns of infection and disease of viruses in a heterologous species remain unpredictable. The One Health model recommends intensifying the efforts in the study of animal pathogens to improve animal health and welfare and ensure animal conservation. This approach strongly applies to endangered animal species such as Iberian lynx.
